# Ethnic Groups Differences in Domestic Recovery after the Catastrophe: A Case Study of the 2008 Magnitude 7.9 Earthquake in China

**DOI:** 10.3390/ijerph14060590

**Published:** 2017-06-02

**Authors:** Ying Wang, Yingqi Zhu, Qi Sui

**Affiliations:** 1 Key Laboratory of Environmental Change and Natural Disaster of Ministry of Education, Beijing Normal University, Beijing 100875, China; Zhuyingqi@mail.bnu.edu.cn (Y.Z.); suiqi@mail.bnu.edu.cn (Q.S.); 2 Academy of Disaster Reduction and Emergency Management, Beijing Normal University, Beijing 100875, China

**Keywords:** ethnic group, households, domestic life recovery, logistic regression, earthquake

## Abstract

This research examined the ethnic differences in domestic recovery after the 2008 Wenchuan Earthquake in China. In 2014, 866 valid questionnaires were collected. Han and Qiang & Zang households were analyzed using logistic regression to determine the factors influencing household recovery. It was found that the householder of the Qiang & Zang group played a more important role in household recovery. Different from the Han, females from Qiang & Zang households had negative attitudes on recovery, and Qiang & Zang households did not believe in the effectiveness of public donations for post-quake recovery. The study also showed that local workers in a household were more helpful for household recovery than were migrant workers in a household, regardless of ethnicity. Therefore, the government should create more local jobs in Han and Qiang & Zang households and pay more attention to women in Qiang households. Assistance should be established specifically for the psychological recovery of Qiang women and family recovery projects.

## 1. Introduction

After natural hazards, inequalities in populations in terms of long-term recovery are evident [1]. The behaviors of people who face natural hazards are influenced by factors including culture, society, and the economy [2,3,4]. 

Hazard vulnerabilities vary according to the type of hazard, as they are contingent on various circumstances (social, psychological, economical, etc.) [5] and unevenly distributed across individuals, households, and regions [6]. Domestic recovery trajectories depend on household internal characteristics, such as household size, income, ethnicity etc. [7,8].

Studies on post-natural hazard recovery often emphasize that people from different ethnic backgrounds are affected differently when struck by a hazard. Understanding radicalized social processes requires a historically informed understanding of the particularities of racial formations in specific places and times, and how these shape the environmental risks to which people are exposed [9,10,11]. Historically and spatially informed research such as Peacock’s work [12] should serve as a model for studies that combine qualitative and quantitative techniques to examine the ways that class, race, and ethnicity shape vulnerability to hazards. 

Scholars have attempted to determine the reasons for these differences based on the relevant backgrounds of race and ethnicity, as different races and ethnicities take different actions towards hazards [13,14]. Generally, racial minorities (mostly people who are black) are more vulnerable than people who are white during post-hazard restoration [15,16,17]. Elliott and Pais [18] collected and analyzed information including recovery time, housing conditions, and the employment status of 1200 survivors of Hurricane Katrina. According to the results, people from different ethnicities responded differently to post-hazard reconstruction. Among these, low-income, black homeowners from New Orleans were more willing to work in their original places of residence. Studying employment recovery after the hurricane, Zottarelli [19] confirmed Elliott’s argument that people who are black from New Orleans were at a particular recovery disadvantage. Zottarelli suggested considering human capital to explore additional reasons when discussing racial differences.

Ethnicities [20] and religions [21] shape our concerns and actions, and in many cases, are why strategies to reduce hazard risk or factors that increase hazard risk are not recognized by society, as they are so embedded in everyday life. Fundamentally, different cultures appear in different ethnicities and races, and cultures significantly affect how ethnic groups cope with the post-hazard reconstruction stage [22]. Therefore, there are significant differences between the ways ethnic groups cope with the post-hazard reconstruction stage. However, does this apply to China or other countries as well? The ethnicities of some countries are classified according to states or regions [2,18,19]. In China, the national attribute is not assigned by the state. In a county there are several ethnic groups, and in the village there will be ethnic settlements. We are interested in knowing whether the recovery of different ethnic groups in China differs.

Social capital is important to post-hazard recovery. Aldrich highlights the critical role of social capital [23] and networks [24] in hazard survival and recovery, and lays out recent literature and evidence on the topic. Social capital is commonly viewed as positively affecting hazard resilience [25]. Kim [26] empirically estimated the association between social capital and natural hazards using Florida counties as a case example, and found that coastal counties with stronger economies and better social conditions before the hazard experienced lower hazard losses.

Economic status in a household is also critical to post-hazard recovery. Homes in low-income and minority neighborhoods recovered more slowly [27]. Schultz [28] used the Spatial Hazard Events and Loss Database for the US and population censuses from 1990 to 2000, finding that income inequality between those in the upper half of the local income distribution and those in poverty tended to increase during hazard recovery. Peacock [29] reported empirical work from Hurricane Andrew in Miami-Dade (FL) in 1992 and the 2008 Hurricane Ike in Galveston (TX) to assess long-term trends in the housing recovery of different households. The findings suggest that social vulnerability factors play out differently in different settings. In Miami, income, race, and ethnicity were critical determinants, whereas in Galveston, income was the more critical factor. Wang [30] researched post-hazard recovery after the 2008 Wenchuan Earthquake, determining that long-term unemployment and poor economic conditions were unfavorable to household recovery. 

According to a comparative post-hazard study, attention should be paid to women’s coping strategies in risky environments based on their characteristics [31,32]. The International Labor Organization working paper on gender, work, and hazard [33] observed that working-class women dependent on social protection, secure employment, public services, and home-based livelihoods were more severely impacted by hazards than men. In addition, most studies found that women are more vulnerable than men when confronting natural hazards [15,34,35]. Moreover, people with a higher education level [1] recover at a quicker pace after a hazard. Investment in education also requires financial support in a family [36]. Therefore, we also want to find out if educational investment is needed for household recovery after an earthquake. 

Little literature explores ethnic household differences in China’s post-hazard recovery. In this study, we are interested in knowing whether the recovery of different ethnic groups in China is different. We focused on ethnic households to explore factors such as gender, age, education, income source, and other household characteristics that affected natural hazards recovery. In our study, a household is defined as those members living under the same roof who make up a distinct economic unit.

## 2. Data Source

The 2008 Wenchuan Earthquake measured 7.9 magnitude and occurred on 12 May in Sichuan province. It caused 69,277 deaths, 374,643 injuries, and 17,923 missing people. After the Wenchuan earthquake, 30,000 Qiang, 10% of the Qiang population, perished [37]. Less than 150 kilometers from the epicenter, most Qiang villages in Beichuan were buried or otherwise devastated [38].

China is a country with multiple ethnic groups, including the majority Han and minorities such as the Qiang, Zang and others. Approximately 91.5% of people in China are Han [39] and are distributed countrywide. Sichuan, one of the western provinces with a large population, is also multi-ethnic. Ethnicities in the province include Han, Qiang, Yi, and Tibetan. 

The Qiang are an old ethnic group in Western China, and the predecessors of the Han, an important part of the Chinese nation [40]. Unlike the Han, most Qiang are mountain dwellers [41,42]. Our study area includes Wenchuan and Beichuan Counties, which are inhabited by the Qiang. The Qiang religion is still at a stage of primitive polytheistic religious belief, worshipping objects such as white stones. Ancestor worship by the Qiang is also common [43]. 

The Qiang are composed of essentially monogamous patriarchal households, and every household is a basic unit of production and life. The family concept for the Qiang is inveterate: the family system prevails in Qiang ethnicities [44], and procreation in the family is strongly influenced by ancestor worship [45]. In closed Qiang villages, the original character of the religious culture and traditional social customs are maintained, especially their social perception of ethnic groups [46]. For the Qiang, the interest of the tribal group is far higher than that of the individual [47].

Unlike the Qiang preference for living in the same geographical region, six waves of large-scale Han migration occurred during their history in Sichuan [48]. Due to freely migrating individuals and encouragement from the Qing government, many Han settled in Sichuan, and the ethnic composition in the area changed dramatically during the Qing Dynasty [49]. Compared to the Qiang, the Han in Sichuan easily accept migration, and it is not expected that family members live in the same region.

After the earthquake, the Chinese government, with the help of the whole country, adopted the partner assistance policy, which has helped in the reconstruction of hazard areas. We assumed that different ethnicities received similar amounts of government aid after the earthquake and that recovery was similar across different ethnic groups.

The researcher surveyed the affected households before the Spring Festival in 2014, finding that most had already completed their housing recovery. All the respondents were adults aged from 18 to 70 years, who survived the Wenchuan Earthquake in Sichuan. The study areas were Beichuan County and Wenchuan County, which were the most affected areas, with a seismic intensity scale of XI. In addition to the Han ethnic group, Qiang & Zang ethnic groups, also live in both of these counties. According to statistics from the end of 2014 [50,51], the population of Beichuan was 238,683 people, comprised of 62.29% Han and 35.80% Qiang. The population of Wenchuan was 99,949, composed of 41.00% Han and 37.40% Qiang. Table 1 shows the ethnic statistics of Beichuan and Wenchuan in 2014.

The survey occurred 10 days before the Spring Festival in 2014 (17 to 25 January). Many migrant workers returned home to be with their families for the festival, which ensured higher sample numbers and better survey efficiency.

Our point-survey was based on simple random sampling. First, according to the intensity of the earthquake and demographic situation of the villages and towns, we determined that the villages and towns with large populations are located in high-intensity areas. The investigators randomly selected households for the survey. In every survey location, the investigators selected respondents randomly based on the house number. In principle, investigators attempted to ensure that the surveyed families reflected the basic situation of the study area. According to the previous investigation and the results of this survey, few households had moved in or out of the area. Therefore, it was possible to determine household situation before and after the hazard could be determined. 

In addition to the research team members, well-trained Sichuan college students also conducted surveys. The model of “investigators inquire-respondents answer” was adopted for the survey, and the questionnaire took approximately 30 min to complete. To unify the criteria of the questions, the investigators were trained before the survey. All respondents were adults aged from 18 to 70 years, as mentioned.

To guarantee the validity of the survey responses with regard to recovery time, investigators first asked household members whether they had moved into new housing, whether the necessities of life had been restored, and the length of time spent on recovery. Respondents were then asked to evaluate whether they had recovered from the hazard and to give a detailed recovery timeline. If the timeline was contradictory, respondents were asked to recall it again. The recovery time for living is the specific time taken to recover all the necessities of life. The unit of time was based on one month, because the hazard occurred a long time ago. 

In the survey, 878 questionnaires were administered. Investigators each house for a face-to-face investigation and collected all questionnaires; thus, the number of valid questionnaires was high. There were 866 valid questionnaires and only 12 invalid questionnaires. Questionnaires were considered invalid when respondents unwilling to answer the questions provided incomplete information. 

The study investigated the factors influencing recovery rates of different ethnic group classifications. The ethnic groups were obtained directly using the 866 valid questionnaires. In China, especially in the area under investigation, there is less intermarriage between different. Therefore, most household members are the same ethnicity as the householder. The questionnaires used in this study were based on the actual situation. According to responses regarding the ethnicity of the householder, the questionnaire was classified into two categories. One is the Han group, the other is the Qiang & Zang group. Ultimately, the Han group completed 422 valid questionnaires, and the Qiang & Zang group 444 valid questionnaires.

## 3. Measures

### 3.1. Dependent Variables

Our two dependent variables represent two levels (the fast and slow) of recovery time after an earthquake. In this research, the household recovery rate was evaluated according to the time spent on recovery efforts. It was presumed that the shorter the recovery time, the better the recovery conditions. The recovery time was based on the survey question, “How long did it take before daily life returned to the same as before? On XX month and XX year.” The recovery time (in units of months) was obtained via calculations beginning in May 2008. According to the recovery time distribution, the households were divided into two categories, namely a fast group and a slow group. Using “average time + standard deviation” as the standard time, it was revealed that households in the fast group spent less than the standard time to recover, while the slow group spent more than the standard time.

### 3.2. Independent Variables

Based on the questionnaires, the current study utilized 8 indexes as independent variables to investigate their relationship with the rate of post-hazard recovery. One numerical variable was the age of the respondent. The seven classified variables were as follows: the gender of the respondent, whether educational investment is needed, whether the householder is always at home, the main source of household income, the change in the total number of temporary workers after the hazard, the change in income after the hazard, and the degree of satisfaction with public donations. 

Respondents’ gender (Male = 1, Female = 0), age and whether the householder is always at home (Yes = 1, No = 0) were obtained from basic information in the questionnaires. Whether educational investment is needed was measured based on whether there was a dependency burden for a juvenile in a household, which indicates that educational investment is needed every year if any children in the household attend school. According to the number of students going to school, in the questionnaire, the index is 1 if the number of students is more than 1, otherwise 0.

Based on the results of the pre-survey, the income of most rural households in this area relies on temporary work. However, the place of work influences income stability differently. According to the results for the question, “What is the main source of income in your household? (ranking)”, the main sources of income are: “1: others (not from working)”, “2: local workers”, and “3: migrant workers”. Compared with household income before the hazard, an increased income was recorded as “1”, decreased income as “2”, and invariant income as “3”. The evaluation of the change in the total number of temporary workers after the hazard is the same as the change in household income. In addition, according to the “the main source of household income” index, household income that does not come from working is separated and recorded as “0” (others), which eliminates confusing factors in the results. Respondents were asked to indicate the degree of satisfaction of each item on a five-point Likert scale ranging from “1: Indifference” to “6: Very Satisfied”. Table 2 shows the statistics of the 7 indexes for the Han and Qiang & Zang ethnic groups.

## 4. Methodology

### 4.1. Descriptive Statistical Analysis

The distribution of the recovery time for the Han and Qiang & Zang ethnic groups in the questionnaires was analyzed using a control group chi-square test. Based on the distribution of the recovery time for the Han and Qiang & Zang ethnic groups, the recovery rate of the two groups was classified into two categories, namely fast and slow.

**Figure 1:**
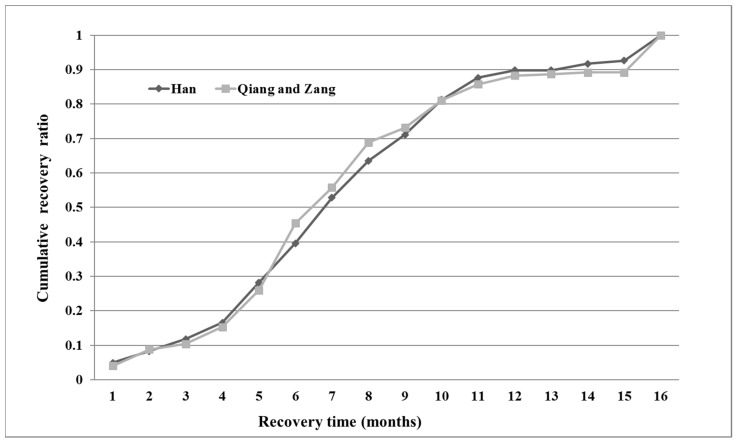
Recovery curves of Han and Qiang & Zang.

Figure 1 traces the summation curve of the recoveries of the Han and Qiang & Zang ethnic groups. The result of the chi-square test for the recovery times of the Han and Qiang & Zang ethnic groups was *p* < *0.01* which indicates significant differences between the two groups. According to Figure 1, during the early period of recovery (January to May), the conditions of the two groups were similar. However, the recovery of the Qiang & Zang ethnic groups was faster than that of the Han during the middle recovery period (May to September). Moreover, during the last period, the two groups were consistent, and the Han ethnic group was somewhat faster than the other group. The factors influencing post-quake recovery of the Han and Qiang & Zang ethnic groups and differences between the factors are analyzed in the following section.

According to the recovery time distribution, the households were divided into two categories. Using “average time + standard deviation” as the standard time, households in the fast group spent less than the standard time recovering, and the slow group spent more than the standard time. Therefore, for Han households (average: *7.701*, standard deviation: *3.756*), a recovery time from 1 to 12 months is recorded as “1” and a recovery time from 12 to 16 months as “0”. The recovery time for the Qiang & Zang households (average: *7.702*, variance: *3.881*) was recorded in the same way. 

### 4.2. Logistic Regression Analysis

The logistic regression model can be expressed by the equation below:

(1) $$P=\frac{1}{1+e^{-\left(X_{1}B_{1}+X_{2}B_{2}+X_{3}B_{3}+X_{4}B_{4}+X_{5}B_{5}+X_{6}B_{6}+X_{7}B_{7}+X_{8}B_{8}+X_{9}B_{9}+constant\right)}}$$

P refers to the possibility for fast recovery after the earthquake (fast recovery when P = 1 and slow recovery when P = 0); 

$X_{i}$(*i* = 1, 2… *n*) is the independent variable. $X_{1}$: The gender of the respondent (Female); $X_{2}$: The age of the respondent; $X_{3}$: Whether the householder is always at home (No); $X_{4}$: Whether educational investment is needed (No); $X_{5}$: The main source of household income (Migrant workers); $X_{6}$: The change in the total number of workers after hazard (Invariant); $X_{7}$: The change in income after hazard (Invariant); $X_{8}$: The degree of satisfaction with public donations (Very satisfied).

$B_{i}$ refers to the coefficient of the independent variable $X_{i}$.

Constant is the constant in the logistic regression model. 

The samples of the “0” and “1” households of the Han and Qiang & Zang ethnic groups are shown in Table 3. We found fewer “0” samples than “1” samples. If the sizes of the “0” and “1” samples differ significantly, the final logistic regression model will be defective. According to the unbiased premise of the logistic regression model, the sizes of the “0” and “1” samples should be approximately equal [40]. Therefore, the sample number of “0” households has been extended to be almost the same as the “1” samples. For example, the number of “0” households is 52, which accounts for 1/7 of the number of “1” households. Therefore, the 52 “0” households were copied seven times and put into the model (364). Similarly, the 63 Qiang & Zang households were copied 6 times and put into the model (378). In our logistic model, the number of “0s” in the Han group is 364, and there are 370 “1s”. The number of “0s” in Qiang & Zang group is 378, and 381 are “1s”.

Table 4 shows the influence of the 8 independent variables obtained through a logistic regression for the post-hazard recovery rate of the Han ethnic group. Table 5 shows the results of the Qiang & Zang ethnic groups. When the cutoff value is 0.5, the accuracy of the model for the Han ethnic group is 78.10% (*p* < *0.01*), whereas the accuracy for the Qiang & Zang ethnic group is 73.90% (*p* < *0.01*). The model used in this study provided favorable predictions for the Han and Qiang & Zang ethnic groups.

In Equation (1), the relation between *X_i_* and the household recovery rate after the earthquake can be judged based on whether its corresponding *Sig.* passes significance testing. Usually, when *Sig. < 0.10*, it has passed the significance test. In addition, * *p* < *0.10*, ** *p* < *0.05*, and *** *p* < *0.01* mean an enhancement of significance testing. 

## 5. Results

In comparing Table 3 and Table 4, the common factors influencing the Han and Qiang & Zang ethnic groups can be observed. (i) When the respondent is older, more time is needed for recovery. The coefficients of the age of the Han and Qiang & Zang ethnic groups are *−0.028* and *−0.030*, respectively, which pass the significance level test at 0.01; (ii) The households whose major income source is from local workers recover faster than those whose major income source is from migrant workers. When the income source from migrant workers is the reference classification, the coefficients of the Han and Qiang & Zang ethnic groups are *1.337* and *0.473*, respectively, passing the significance level test at *0.01* and *0.05* respectively. The result is similar to the post-quake survey in Yunan because migrant workers spend only part of their income on local household construction, and must spend another part of their income at their place of work [52]; (iii) Households with increased income after the earthquake recover faster. The reference classification is “the income is invariant”. If income increased, the coefficient is positive (Han ethnic group: *0.434*, Qiang & Zang ethnic groups: *0.510*); otherwise, the coefficient is negative (Han ethnic group: *−0.660*, Qiang & Zang ethnic groups: *−0.749*); (iv) Whether a household must invest in education makes little difference for post-hazard recovery. When the reference classification is “a household needs educational investment”, both the *Sig.* of Han and Qiang & Zang are approximately *0.100*.

In addition, the variables with large differences in significance levels or coefficients in the logistic regression model between the Han and Qiang & Zang ethnic groups were compared. First, males from a Qiang & Zang household evaluated household recovery higher. In the logistic regression, the variable “the gender of the respondent” for the Han ethnic group failed to pass significance testing (*t* = *−1.447*, *p > 0.5*), but this variable for the Qiang & Zang ethnic groups passed (*t* = *2.908*, *p* < *0.01*). Therefore, the gender of a respondent will influence the self-evaluation of the household recovery rate in a Qiang & Zang household. When the reference classification is female, the coefficient is 0.538, which indicates that a male from a Qiang & Zang household will evaluate household recovery higher. However, respondents from Han households showed no differences.

The “satisfaction degree with public donations” also differs between the two groups. Using “very satisfied” as the reference classification, this variable passed the significance test (*p* < *0.01*) in Han households with a positive coefficient, which indicates that the households with a higher degree of satisfaction recover faster. Regarding the Qiang & Zang households, although the variable passed the significance test (*p* < *0.01*), the significance levels of the different categories are relatively poor (three are *p > 0.1*, and two are *0.01* < *p* < *0.1*). This difference indicates that Han households believe in the effectiveness of public donations for post-quake recovery, but that Qiang & Zang households do not. On the night of the quake, the Chinese State Council organized an emergency meeting and the General Headquarters of Quake Relief was immediately estableshed within the State Council to coordinate rescue and relief efforts [53]. After the earthquake, the Chinese government encouraged public donations. Furthermore, Yamamura [54] found a positive relationship between social trust and happiness after the Great East Japan earthquake. In general, the families under investigation were satisfied with public donations after the Wenchuan earthquake in China.

The variable “whether the householder is always at home” reflects different values. Utilizing “the householder is not always at home” as the reference classification, this variable of the Han households fails to pass the significance test (*t = 0.751, p > 0.10*), while the variable of the Qiang & Zang households passes the significance test (*t = 2.761, p < 0.01*) with a coefficient of 0.588. This result indicates that whether the householder is always at home makes no difference in household recovery in the Han households, but it is favorable for the Qiang & Zang households.

Finally, we focused on the change in the total number of workers after the hazard. Using “the total number of workers is invariant” as the reference classification, the variable for Han households passed the significance test. The coefficient for non-temporary working households is *1.695* (*t = 4.632, p < 0.05*). When the number of workers increases, the coefficient is *−0.504 (t = −2.152, p < 0.05)*; otherwise, the coefficient is *1.067 (t = 2.100, p < 0.05)*. This result indicates that income not obtained from temporary workers in Han households negatively influences recovery, and an increase in the total number of these households negatively influences household recovery. However, for Qiang & Zang households, this variable fails to pass the significance test *(p > 0.1)*.

## 6. Discussion

In previous studies, education was widely accepted as an indicator of an individual’s earthquake hazard perception [55,56,57,58]. In addition, it has been shown that people with a higher level of education were less likely to have a post-traumatic stress disorder [59,60,61]. In general, survivors with higher education levels had a better ability to overcome difficulties caused by an earthquake; therefore, the recovery time was shorter [62].

This study also found that education levels had little significance in post-hazard household recovery. Among the survey samples, 68.40% of individuals had an education level lower than primary education. It was concluded that, because there was little difference in residents’ education, education was not significant in the recovery analysis.

Han and Qiang & Zang households who still had to invest in education demonstrated slower hazard recovery (*−0.367,* *p < 0.10; −0.302, p = 0.10*). If any school-aged children were present, persistent education investment from households was expected. According to the field survey, 45.60% Qiang & Zang and 31.60% Han households were investing in education. Educational investment becomes a small burden to households during earthquake recovery. The government should plan to provide funds for elementary education after the earthquake, not just relief materials.

Although Han and Qiang & Zang people in hazard-stricken regions shared the same natural environment and, governmental and social support, and were deeply rooted in culture, education and customs, there were generally few significant differences in their post-hazard recovery processes. In Qiang & Zang households, females evaluated their household recovery as being slower. The “satisfaction degree with public donations” in Qiang & Zang households was more optimistic. The householder at home was favorable for the recovery of Qiang & Zang households. In addition, the change in the total number of temporary workers after the hazard had no significant effect on Qiang & Zang households. Furthermore, the main source of household income in both the Han and Qiang & Zang ethnic groups was critical for household recovery. Local workers in a household were more helpful to household recovery than migrant workers in a household, regardless of ethnicity. 

The government should pay more attention to the women in Qiang households, and assistance should be estableshed specifically for the psychological recovery of Qiang women and family recovery projects, because they are more pessimistic about the recovery of their own families. The householder in Qiang has a very important role in recovery; therefore, the government should initiate assistance projects for householders in Qiang to help them with local employment. At the same time, the government should create more local jobs in both Han and Qiang & Zang households, so that they have a sustained source of income, rather than having to rely on short-term capital assistance.

There are some shortcomings in this paper. Social capital variables are important factors affecting recovery. However, because of the large sample survey, this part of data acquisition was more difficult; thus, this article does not discuss the impact of social capital on post-hazard recovery. In addition, the recovery time in this study was based on the recall of respondents, and because the period of recovery was long, there may be some deviation. Therefore, more research should be conducted in the future to keep track of the survey. 

## 7. Conclusions

This research provides new evidence regarding the differences and similarities in domestic recovery between different ethnic groups through field surveys, which complement the experimental evidence in related literature. 

The study revealed common influence factors as well as differences in the household recovery process of Han and Qiang & Zang ethnic groups following the 2008 Wenchuan Earthquake. In this research, a descriptive statistical analysis indicated a similar recovery process for Han and Qiang & Zang households. Finally, by analyzing the factors for the two groups’ recovery processes through a logistic regression model, the study found no differences between some factors influencing the recovery process for the Han and Qiang & Zang groups. Older populations needed more time for recovery after the earthquake. The households whose major source of income was from local workers recovered faster, as did households with increased income after the earthquake.

In recent decades, the Han and Qiang & Zang populations have become deeply involved in the process of urbanization in Sichuan, and the ethnic groups are moving forward in harmony. After the earthquake, the main source of household income for both the Han and Qiang & Zang ethnic groups was significant in household recovery. As land destroyed in the earthquake was occupied for rebuilding, the main source of post-hazard income for households in both groups was temporary work. Moreover, households where major income was from local workers after the hazard recovered much faster than those whose major income was from migrant workers. It is worth noting that local workers in a household were more helpful to household recovery than migrant workers in a household, regardless of ethnicity. 

In addition, we should also examine the differences in post-hazard recovery of the two ethnic groups. A male from a Qiang & Zang household evaluated household recovery higher. However, respondents from Han households showed no differences. Previous papers identified gender as a very important factor in determining behavior and recovery after a natural hazard such an earthquake. The Qiang & Zang households followed that conclusion, because women have additional dependencies and household responsibilities, and face greater risks in hazards compared to men in a household [63].

Householders played a critical role in the post-hazard recovery process for Qiang & Zang households. In other words, Qiang & Zang households relied on their householder more. For example, in the highly patriarchal Qiang society, people think highly of their own family. As described in the overview of ethnic, Qiang people sharing clan ancestors are firmly linked by blood relationships and live in the same geographical region. However, the Han people have been in a state of high mobility throughout history. The Han easily accept migration, and family members are not necessarily expected to live in the same region. Compared to Qiang & Zang males, female members in Qiang & Zang households were more likely to hold an optimistic attitude towards their post-hazard recovery. However, respondents from Han households showed no differences. Furthermore, the Han respondents believed that donations were strongly favorable to the recovery of their households, whereas the Qiang & Zang respondents did not. 

## Figures and Tables

**Table 1:** Ethnic Statistics of Beichuan and Wenchuan in 2014.

	Han	Qiang	Zang	Others
Beichuan	62.29%	35.80%	1.50%	0.41%
Wenchuan	41.00%	37.40%	20.10%	1.50%

**Table 2:** Summary Statistics.

Category and Variable	Han	Qiang and Zang	Survey Questionnaire
**Gender**			**What is your gender?**
Male	55.90%	48.00%	1. Male
Female	44.10%	52.00%	2. Female
**Household at home**			**Does the householder stay at home all year round?**
Yes	73.40%	68.50%	1. Yes
No	26.60%	31.40%	2. No
**Education**			**The number of people in a household who go to**
Above primary school	31.60%	45.60%	1. Primary school 2. Junior high school 3. Senior high school, or university and above.
Primary school	68.40%	54.40%	
**The main source of the household income**			**What is the main source of income in your household? (ranking)**
The main income of the household is not from working, but other sources	19.81%	16.89%	1. Planting 2. Cultivation 3. Transportation 4. Local working
Working inside	51.05%	44.14%	5. Migrant working 6. Government subsidy
Working outside	29.14%	38.96%	
**Total working population after the earthquake**			**How many household members went to work before/after the earthquake?**
The main income of the family is not from working, but others.	51.10%	55.30%	
Increase	22.30%	18.80%	
Decrease	7.30%	7.20%	
The same	19.30%	18.60%	
**Income after the earthquake**			**How has your household income changed in comparison to before the earthquake?**
Increase	34.40%	41.00%	1. Increase
Decrease	42.50%	39.50%	2. Decrease
The same	22.50%	19.00%	3. The same
**The degree of satisfaction with government aid**			**Are you satisfied with government aid?**
Very satisfied	8.10%	6.30%	1. Very satisfied
Satisfied	27.10%	37.50%	2. Satisfied
Not bad	21.10%	20.90%	3. Not bad
Dissatisfied	21.50%	29.50%	4. Dissatisfied
Very dissatisfied	20.10%	5.30%	5. Very dissatisfied
Indifferent	1.80%	1.10%	6. Indifferent

**Table 3:** Distribution of Degree of Recovery.

Ethnic	Strong Recovery	Weak Recovery
Han	360	52
Qiang and Zang	381	63
Classification value	1	0

**Table 4:** Details and tests of multiple predictor logistic regression model in Han households.

Variables	B	S.E.	Wald	df	Sig.	Exp(B)
The gender of the respondent (Female)	−0.306	0.211	2.095	1	0.148	0.736
The age of the respondent	−0.028 ***	0.008	11.870	1	0.001	0.972
Whether the householder is always at home (No）	0.196	0.261	0.564	1	0.453	1.217
Whether educational investment is needed (No)	−0.367 *	0.220	2.797	1	0.094	0.693
The main source of household income (Migrant workers)	***		33.992	2	0.000	
Others (not from doing temporary work)	1.659 ***	0.358	21.455	1	0.000	5.256
Local workers	1.337 ***	0.259	26.566	1	0.000	3.806
The change in the total number of workers after disaster (Invariant)	***		37.053	3	0.000	
The main source of income is not from doing temporary work, but other sources	1.659 ***	0.358	21.455	1	0.000	5.256
Increase	−0.504 **	0.234	4.630	1	0.031	0.604
Decrease	1.067 **	0.508	4.409	1	0.036	2.907
The change in income after disaster (Invariant)	***		20.254	2	0.000	
Increase	0.434	0.297	2.136	1	0.144	1.543
Decrease	−0.660 **	0.282	5.476	1	0.019	0.517
The degree of satisfaction with public donations (Very satisfied)	***		133.137	5	0.000	
Indifferent	−5.994 ***	1.312	20.884	1	0.000	0.002
Very dissatisfied	−6.392 ***	0.850	56.608	1	0.000	0.002
Unsatisfied	−2.877 ***	0.755	14.535	1	0.000	0.056
Ordinary	−3.582 ***	0.757	22.397	1	0.000	0.028
Satisfied	−1.841 **	0.757	5.907	1	0.015	0.159
Constant	4.135 ***	0.914	20.465	1	0.000	62.494

* Sig. < 0.10, ** Sig. < 0.05, *** Sig. < 0.01.

**Table 5:** Details and tests of multiple predictor logistic regression model in Qiang and Zang households.

Variables	B	S.E.	Wald	df	Sig.	Exp(B)
The gender of the respondent (Female)	0.538 ***	0.185	8.455	1	0.004	1.713
The age of the respondent	−0.030 ***	0.007	16.799	1	0.000	0.970
Whether the householder is always at home (No）	0.588 ***	0.213	7.621	1	0.006	1.801
Whether educational investment is needed (No)	−0.302	0.184	2.700	1	0.100	0.739
The main source of household income (Migrant workers)	*		5.607	2	0.061	
Others (not from doing temporary work)	21.471	4731.419	0.000	1	0.996	2,111,437,206.785
Local workers	0.473 **	0.200	5.607	1	0.018	1.604
The change in the total number of workers after disaster (Invariant)			3.465	3	0.325	
The main source of income is not from doing temporary work, but other sources	−42.371	7159.926	0.000	1	0.995	0.000
Increase	−0.355	0.191	3.440	1	0.064	0.701
Decrease	−0.148	0.332	0.199	1	0.655	0.862
The change in income after disaster (Invariant)	***		38.149	2	0.000	
Increase	0.510 **	0.241	4.469	1	0.035	1.665
Decrease	−0.749 ***	0.248	9.150	1	0.002	0.473
The degree of satisfaction with public donations (Very satisfied)			22.513	5	0.000	
Indifferent	0.651	18,589.919	0.000	1	1.000	1.918
Very dissatisfied	0.038	0.524	0.005	1	0.943	1.039
Unsatisfied	−0.257	0.385	0.447	1	0.504	0.773
Ordinary	−0.893	0.406	4.832	1	0.028	0.409
Satisfied	0.281	0.371	0.576	1	0.448	1.325
Constant	1.092	0.550	3.943	1	0.047	2.981

* Sig. < 0.10, ** Sig. < 0.05, *** Sig. < 0.01.

## Supplementary Materials

Supplementary File 1
